# Open Air Quality Data Platforms for Environmental Health Research and Action

**DOI:** 10.1007/s40572-025-00487-6

**Published:** 2025-08-01

**Authors:** Colleen Marciel Rosales, Jennifer R. Bratburd, Sebastian Diez, Sara Duncan, Carl Malings, Pallavi Pant

**Affiliations:** 1 OpenAQ, Washington, DC USA; 2 Air Quality Research Center, University of California, Davis, CA USA; 3 University of Wisconsin, Madison, WI USA; 4 Centro de Investigacion en Tecnologias para la Sociedad, Universidad del Desarollo, Santiago, Chile; 5 School of Health Sciences, Western Carolina University, Cullowhee, NC USA; 6 Morgan State University, Baltimore, MD USA; 7 NASA Goddard Space Flight Center, Greenbelt, MD USA; 8 Health Effects Institute, Boston, MA USA

**Keywords:** Open Data, Air Quality, Air Pollution, Air Quality Databases, Air Quality Repositories, Satellite Remote Sensing Data, Ground-level Monitoring, Air Sensors

## Abstract

**Purpose of Review:**

Air pollution remains the greatest environmental threat to public health, and significantly impacts many aspects of human life. This review surveys digital platforms that provide data openly and make air quality data available for use and analysis by environmental health researchers for scientific research and to inform air quality action.

**Recent Findings:**

Successful programs that rely on different types of open air quality data have been observed and exist as models for regions that have yet to improve their air quality. However, disparities remain in the availability and granularity of generated air quality data in resource-rich and resource-poor regions. Even if air quality data are made available and open, their usability and actionability still remain challenging. Determinants of usability may include the user’s technical know-how and ability to deal with disparate data; compute capabilities; barriers in data sharing and data generation; and misuse and misclassification of data and levels and sources of exposure. However, the synthesis of different sources of data and advancements in modeling may help fill in spatiotemporal gaps in many areas of the world. In addition, the democratization of air quality data is facilitated by advancements in air quality data generation (e.g., data collection by open-source air sensors) and the ability to contribute to global, open-access, publicly available repositories that openly share data.

**Summary:**

Many open data platforms exist for sharing and accessing ground-based/in-situ data, satellite remote sensing data, and modeled or otherwise derived outputs. Online platforms that allow visualization and analysis without the need to download software are also now widely available. Despite wide availability, disparities in accessibility exist—expansion and support through interdisciplinary collaboration and continuous funding support for air quality data is essential for the continuous global benefit and community-specific action to address air quality as an environmental health issue.

## Introduction

Air pollution remains the greatest environmental threat to public health, and significantly impacts well-being, economy, trade, food security, and many other aspects of human life [1]. While the world has experienced an average slight decline in air pollution in recent years, the burden due to non-communicable diseases resulting from air pollution is high and growing in many regions worldwide [2]. In particular, populations from low- and middle-income countries (LMICs) are exposed to 1.3–4 times higher levels of ambient particulate matter (PM_2.5_) than those in high-income countries [2]. LMICs may lack air quality monitoring programs, national air quality standards, and other critical analysis tools to assess pollution emissions and impacts on health [3–5]. For example, a report by the United Nations Environment Programme (UNEP) in 2021 stated that 34% of countries do not have protections against ambient air quality and 86% of these countries do not have air quality standards at all [6]. In 2025, the World Health Organization (WHO) released an *Air Quality Standards database* (https://www.who.int/tools/air-quality-standards*)*, where it was reported that there are still many countries that do not implement standards for pollutants [7]. In addition, while regional ambient pollution has improved in high-income countries, concentrations can vary drastically at smaller scales [8]. Confounding these issues are threats to federal or national-level monitoring and enforcement programs and possible laxes in regulation that may lead to increased exposures [9].

Inequalities in air pollution exposure correspond with disparities in the technical, computational and geographical capacities required to collect, access, and use air quality data effectively. Globally accessible open data platforms that make air quality information freely available can address some of these disparities. However, many users in resource-limited regions may still lack the computational infrastructure, technical skills, or internet connectivity to work with large, machine-readable datasets, which can hinder their ability to leverage these tools for local decision-making. Addressing the uneven access and ability to utilize large-scale datasets is critical for ensuring that open data platforms achieve their full potential in promoting equitable access to air quality information and action. This review will focus on open data platforms for air quality, particularly those making data universally and widely available for air pollutants identified in the World Health Organization (WHO)’s air quality guidelines, i.e., fine particulate matter (PM_2.5_), coarse particulate matter (PM_10_) nitrogen dioxide (NO_2_), ozone (O_3_), sulfur dioxide (SO_2_) and carbon monoxide (CO) [10].

According to the Open Data Toolkit, data are “considered ‘open’ if anyone can freely access, use, re-use, and redistribute them, for any purpose, without restrictions” [11]. Furthermore, true openness requires making the data available in open formats that software can read (computable and machine-readable), allowing users to readily query and re-use the data; for example, publishing a portable document format (PDF) on a government website is not considered “open.” [12, 13] In the case of air quality data, an example “use case” would be downloading ground-level air quality measurements and feeding them into a machine-learning model that combines these measurements with satellite measurements to create air quality forecasts [14]; such a process would be considered “open” if both ground-level and satellite datasets were freely and publicly accessible, straightforward to use, and searchable. Additionally, machine learning models used must be open-source; as well as the resulting forecast and its metadata, with the code and data products shared in a public repository.

In this work, we first describe the main types of air quality data—ground-based, satellite, modeled, and derived—and how each contributes to environmental health research. We then highlight key platforms and repositories that facilitate access to these data, followed by a discussion on challenges that limit accessibility and use. Finally, we present recommendations to enhance the utility and equity of open air quality data globally.

### Case-Studies

Accessible measurements of air pollution are critical for decision-support applications: for example, having air quality forecasts to help people plan their day to reduce exposure to ambient air pollutants; helping communities, researchers and governments measure exposures to populations to understand and study impacts on health; and evaluating emission sources and effective strategies to reduce emissions. Open data should be used to inform the public, while ideally reducing exposure through changed policies or other actions. This can occur at the continental, country, state, city, municipality or other administrative levels. Often, locally or hyper-locally applied data can help to increase understanding of and involvement in improving air pollution in communities [15]. A variety of open data networks have been used to better understand local air pollution, particularly at the hyper-local level (10 m or less). A well-known example is in the United States (US), where the Clean Air Act has successfully led to the improvement of air quality, with extensive air quality monitoring and the requirement to make the data publicly available. Setting up regulatory networks, as well as informational networks helps continuously inform regulations. For example, the update to the US National Ambient Air Quality Standard for PM_2.5_ from 12.0 to 9.0 µg/m^3^ was informed by long-term air quality data trends as seen from air quality monitoring networks, alongside other data [16]. Likewise, the WHO updated its recommended annual PM_2.5_ guideline in 2021 from 10 µg/m^3^ to 5 µg/m^3^, and for NO_2_ from 40 µg/m^3^ to 10 µg/m^3^. Another example is the *Aires Nuevos* project in which low-cost air sensors were placed throughout cities in Central and South America. The data from these sensors were used for local non-regulatory reporting such as that described by Silva et al. [17].

For air quality forecasts, accessible public data helps improve tools. The US EPA operates the Fire and Smoke Map (https://fire.airnow.gov/*)*, which aims to provide the public with timely, interpretable air quality data as it relates to fires and smoke, such as those from wildfires [18]. The Copernicus Atmosphere Monitoring System (CAMS) provides five-day forecasts for air pollutant concentrations (https://atmosphere.copernicus.eu/charts/packages/cams/*)* for the entire globe. The data have been leveraged for a variety of applications, including an app service for residents and visitors in Greece (https://atmosphere.copernicus.eu/discovair*).*

### Data Types

Data related to air quality can be either quantitative or qualitative. Numerical air quality measurements in physical units (reported in concentration units such as µg/m^3^ for PM_2.5_) as collected by an air sensor or a reference monitor, and associated readings like timestamps (date and time) and geographical coordinates are quantitative measurements. On the other hand, qualitative data may include descriptive or subjective information (e.g., observations of visibility ranked on a Likert scale), reports and anecdotes of symptoms from individuals, narrative descriptions of air pollution sources, and stories of lived experiences of communities. This paper describes quantitative air quality data openly available for use in environmental health research. The use and availability of qualitative data for environmental health research in air quality is outside the scope of this review.

### Ground-Based in-Situ Data

Ground-based, in-situ measurements are key for accurate quantitative characterization of air pollutants. These direct measurements serve as “ground truth” for the calibration and validation of other air quality data (e.g., model outputs, satellite products, sensors, derived data products, etc.). These data may also be “ingested” to enhance the accuracy of models, satellite data, sensors, or derived products. In-situ observations capture fine-scale local and/or short-term variations that are often missed by satellite data or models, making them indispensable for air quality monitoring and research. Table 1 provides some examples of currently existing air monitoring networks.

Although the spatial coverage of ground-based networks is more limited compared to satellites, they remain crucial for understanding air quality in both urban and rural areas. These networks typically support regulatory monitoring and can also be used for exposure assessments and epidemiological studies, particularly in densely populated urban settings. Real-time data also provide actionable insights during pollution episodes, such as wildfires or industrial accidents, aiding emergency responses. In rural areas, ground-based data are particularly valuable for monitoring agricultural pollutants (e.g., ammonia, pesticides, dust), as well as the impacts of transported urban emissions (e.g., O_3_) and emissions from fires.

In LMICs, ground monitoring in rural areas can also be helpful in estimating the localized contributions of household air pollution due to the use of solid fuels for cooking, heating and other residential activities. Emissions from solid fuel use for cooking or heating often disperse into the surrounding outdoor environment, elevating ambient concentrations. In fact, household air pollution has been estimated to contribute to ~ 20% of the global ambient PM_2.5_ [19]. Ground-based monitors located near homes or in village centers can thus provide valuable information about the regional air quality burden from residential energy practices—data that is often missed or underestimated by satellites and coarse-resolution models. These measurements also help assess their effects on sensitive ecosystems, including crops and forests, and provide baseline data to evaluate future changes related to land-use changes or industrial development.

However, ground-based monitoring systems require regular maintenance and calibration to ensure data accuracy and comparability, which can be particularly challenging in resource-limited settings. High costs and limited technical capacity—both in terms of skilled personnel and infrastructure—may pose significant barriers in less advantaged regions, often hindering the geographical coverage, sustainability and effectiveness of these networks [13, 20–22]. Because of these costs and challenges, large-scale air quality networks are typically operated and funded by governments. However, even in resource-rich countries with more dense monitoring networks, gaps in coverage can have policy implications that limit the effectiveness of air quality policies in unmonitored regions, though there is potential to supplement this with other data sources [23, 24]. Strengthening local capacity through training programs is essential for enabling teams to install, maintain, and calibrate these systems, thereby ensuring their long-term viability [25, 26]. Other significant barriers in resource-constrained environments include the difficulty of procurement of spare parts, especially for reference monitors, and frequent power outages that can result in loss of data. For example, between 27 and 33% of data were estimated to have been lost due to load shedding in South Africa [27, 28]. In Nairobi, Kenya, a spare part for the reference-grade ambient PM_2.5_ monitor could not be secured locally, and the instrument had to be shipped outside the continent for repairs; this resulted in a loss of nearly a year’s worth of air quality data [29]. Additionally, the lack of standardized protocols and interoperability among monitoring networks managed by different entities often limits their integration into open data platforms and complicates comparisons across datasets. Also, certain government and private ground-based networks do not make air quality information openly accessible—in 2024, only 28% of national governments share their air quality monitoring data publicly [13]. Addressing these challenges is crucial to maximizing the utility of in-situ networks, filling gaps in areas with sparse geographic coverage, and fostering equitable access to high-quality air pollution data worldwide.

A significant opportunity to complement traditional ground-based networks in regions with limited monitoring infrastructure is the use of emerging “low-cost sensors”. While their accuracy does not match that of reference-grade monitors, their affordability (with respect to reference-grade monitors) and ease of deployment make them a practical choice especially for community-led monitoring to address localized environmental challenges and for monitoring in LMICs. For details on the advantages, limitations, and opportunities afforded by low-cost sensors in relation to other data types, we refer the reader to [30]. In terms of data openness, like other in-situ data, this can vary, with some sensor manufacturers and networks providing open data and access via free online platforms, while others restrict data access or require paid subscriptions. Examples of low-cost sensor networks providing open data are also included in Table 1.

**Table 1:** Some examples of existing air monitoring networks providing open data. While many of these resources provide global-level data, some disparities still exist in the abundance of data between resource-rich and resource-poor areas, resulting in a disparity and incompleteness with respect to geographic coverage.

In-situ reference-grade air quality networks	Capture fine-scale temporal or geographical variations	Limited spatial coverage	EANET [31, 71]	Asia
	Serve as “ground truth” for calibration and validation of other air quality data	Require time- and cost-intensive regular maintenance and calibration	CSN [72]	USA
	Can be collocated with meteorological stations		IMPROVE [73, 102]	USA
			ACTRIS [32, 74]	Europe
			SINAICA [75]	Mexico
			AURN [76]	UK
			SPARTAN [33, 77]	Global
			ASCENT [78]	USA
			RAMA [79]	Mexico City
“Low-cost” sensing networks	Cost of procurement and maintenance of nodes are less expensive relative to reference-grade monitors	Validation and corrections against reference-grade monitors might be necessary	Breathe Cities [80]	14 cities
	Amenable to integration to “smart” technologies	Require regular maintenance and calibration	SIATA [81]	Colombia
			sensor.community [82]	Global
			opensensemap.org [84]	Global
			verpm [83]	Mexico
Satellite remote sensors & missions	Can provide data at regional to global scales, covering more area than local networks	Unable to measure directly measure near-surface pollutant concentrations	MODIS [85]	Global
	Complements ground-based measurements by capturing spatial patterns not observable by in-situ measurements	Difficulty of measurements at specific conditions (e.g., nighttime, dense cloud or smoke)	VIIRS [86]	Global
		Unfamiliar, sophisticated data formats can present barriers to accessibility	OMI [87]	Global
			TROPOMI [88]	Global
			GOES ABI [89]	Western
			Meteosat FCI [90]	Hemisphere
			Himawari AHI [91]	Atlantic Hemisphere
			TEMPO [92]	Pacific Hemisphere
			GEMS [93]	North America
				East Asia
Remote sensing ground-based networks	Complement “top-down” information from satellites with “bottom-up” atmospheric information at specific locations	Limited spatial coverage	PANDONIA [94]	Global
	Can help relate satellite data to near-surface concentrations	Require time- and cost-intensive regular maintenance and calibration	AERONET [95]	Global
			EARLINET [96]	Europe
			LALINET [34, 97]	Latin America
			TCCON [98]	Global
			MPLNET [99]	Global
			SHADOZ [100]	Southern Hemisphere
			GAW [101]	Global

Epidemiological and exposure assessment studies have demonstrated the effective use of air quality data from ground-based networks. Some of the earliest studies that established the association between exposure to pollution and poor health outcomes utilized data from ground-based air quality monitoring sites. For example, in the US, air quality data from the Inhalable Particulate Monitoring Network (IPMN) and Aerometric Information Retrieval System (AIRS), which later became the US EPA’s Air Quality System (AQS), was used in multiple studies to show the association between air pollution and respiratory and cardiovascular mortality [35, 36]. Data from ground-based networks has also been used to study the association between short-term exposure to air pollution and various health outcomes, including hospital admissions and mortality. For example, in China, data from the Shanghai Environmental Monitoring Center (SEMC) was used to study the association between coarse (PM_10_) and fine (PM_2.5_) particulate matter and daily mortality [37]. Another study utilized data from more than 600 cities globally and reported an association between short-term exposure to PM_10_ and PM_2.5_ and daily all-cause, cardiovascular, and respiratory mortality [38]. Hayes, et al. showed the relationship of asthma-related emergency hospital admissions in Mexico City from 2017 to 2019 by using fixed-site AQ monitoring stations in different geographical areas across Mexico City from their automatic atmospheric monitoring network, Red Automática de Monitoreo Atmosférico (RAMA), which is hosted by the Mexico City Government [39, 79]. Similarly, studies that correlate hospital visits with air pollution levels from ground-level air quality monitoring data have been conducted in Lanzhou, China and Ho Chi Minh City, Viet Nam [40–42]. Relationships between wildfire and respiratory health have also been investigated using available air quality data from ground-level networks [43, 44].

### Satellite and Ground-Based Remote Sensing Data

Satellite remote sensing is another tool for understanding air quality at regional to global scales. Such sensors routinely collect information on the presence and concentrations of aerosols and trace gases in the atmosphere. The spatial and temporal resolution of these data are continuously improving, allowing better urban-scale analysis and near-real-time information. For example, the European Space Agency (ESA) TROPOMI [88] instrument (launched 2017) provides daily global coverage of up to 5.5 km by 3.5 km resolution for trace gasses like NO_2_ and SO_2_, while geostationary missions like Geostationary Environment Monitoring Spectrometer (GEMS) over East Asia (launched 2020) [93] and TEMPO over North America (launched 2023) [92], and the planned Sentinel-4 mission over Europe and North Africa provide similar information on an hourly basis for their respective regions. Air quality and its health impacts are also becoming a higher priority; the Multi-Angle Imager for Aerosols (MAIA) mission (https://maia.jpl.nasa.gov/) planned for launch in 2026 is the first explicitly public health-focused mission of the US National Aeronautics and Space Administration (NASA), and includes both ground-based and space-based components as well as planned epidemiological studies to better connect aerosol concentrations and properties with their health impacts in several cities across the globe.

Satellite data complements ground-based measurements by capturing spatial patterns and trends not observable using spatially limited in-situ measurements [45]. Satellite data are also suitable for the detection of emission sources (e.g., wildfires and point sources such as oil and gas fields) and the development or validation of emissions inventories using a top-down approach [46]. An inability to directly measure near-surface pollutant concentrations is a notable limitation of satellite data in air quality applications; other information are needed to connect remote sensing data to the near-surface conditions most relevant for human and environmental health. The inability to make certain measurements at night or under dense cloud or smoke conditions can also bias a purely satellite-based analysis toward clear-sky daytime conditions [47]. Thus, while not sufficient on their own, satellite-retrieved data can be used in conjunction with other data to gain a comprehensive picture of air pollution.

Ground-based remote sensing networks represent systems of remote sensing instruments which point up into the atmosphere, providing a “bottom-up” perspective of atmospheric pollutants, which can complement the “top-down” view of satellite remote sensors. Operating from fixed locations, they can provide full daytime (and in some cases overnight) coverage, compared to more limited overpass “snapshots” offered by satellites. Active ground-based remote sensors, such as lidars, can also provide details on the vertical distribution of pollutants in the atmosphere. When paired with ground-based in-situ measurements, they can help establish the relationships between remotely sensed quantities and near-surface pollutant concentrations needed to translate satellite information into quantities more relevant for air quality. However, their spatial distributions are sparser than in-situ ground networks.

Publicly funded agencies that operate satellite missions and ground-based remote sensing networks, such as NASA and ESA, have mandates to make their data open. While commercial satellite data providers are becoming increasingly relevant for optical and radar imagery, they are as yet not prevalent in the provision of data in spectral bands relevant for air quality (although this may change in the future), and the highest quality satellite datasets for atmospheric pollutants remain available cost-free, e.g., through NASA Earthdata or ESA Copernicus. However, there are still barriers to accessibility and usability which limit the openness of satellite data. Large file sizes and unfamiliar, sophisticated data formats and terminology can make these data inaccessible to those without appropriate computational resources and subject matter expertise. The myriad of missions and datasets to choose from can also be overwhelming to new users. These problems, however, have been recognized and there are institutional efforts to address them, e.g., via *NASA’s Open Science Initiative *(https://science.nasa.gov/open-science/tops/), *NASA Applied Remote Sensing Training Program (ARSET*) capacity building (https://appliedsciences.nasa.gov/what-we-do/capacity-building/arset), and *NASA Health and Air Quality Applied Sciences Team (HAQAST*) partnerships with data users (https://haqast.org/).

### Modeling

Modeling via numerical simulation of air quality is important for better understanding the processes affecting air quality and providing context for observations. It can also be useful for forecasting and prediction that enables protection and informs action. Atmospheric composition models, such as zero-dimensional box models, transport models, or global three-dimensional (3D) models are particularly useful for forecasting future air quality and for running counterfactual simulations to investigate policies aimed at improving air quality. Creating a realistic model simulation, however, requires information on historical and/or projected emissions of various pollutants. Observational data are also required to validate model performance. Finally, observational data can be used to update and adjust models to bring them more in line with real-world conditions via data assimilation. In all cases, the open availability of these input data would facilitate their use. Open provision of modeling results is also an important consideration to support open science (e.g., even those without the resources or expertise to operate the models can still analyze the outputs); examples of openly available outputs include those of the Goddard Earth Observing System Composition Forecast and European Centre for Midrange Weather Forecasting CAMS Forecast, as mentioned in the previous section “Case Studies”. Open model architectures (e.g., those based on open-source software and allowing for community involvement and updating, such as the Community Multiscale Air Quality Modeling System) and open modeling platforms (e.g., accessible computational resources such as cloud computing) are further dimensions to consider regarding the openness of modeling.

### Derived Datasets

Derived datasets synthesize other air quality information, such as in-situ measurements and satellite remote sensing data, using numerical and/or statistical modeling, often with the goal of producing spatially and temporally complete datasets. Creating derived datasets can also reduce biases and uncertainties versus a single information source, especially in the case of relating satellite and/or atmospheric model outputs to surface-level pollutant concentrations and air quality. A common example is land use regression models, which use spatial characteristics of land use and cover to extrapolate from sparse in-situ measurements to spatially complete exposure maps. In many cases, derived datasets are customized and adapted to a specific study domain, time interval, and objective to maximize their local applicability and relevance in a specific use case, at the expense of generalizability. There are also more explicitly general datasets, such as global atmospheric composition reanalyses (e.g., https://disc.gsfc.nasa.gov/datasets?page=1%26measurement=Particulate%20Matter%20(Pm%202.5)%26project=MERRA-2, *CAMS Reanalysis *[103]) and derived datasets for PM_2.5_ (e.g., https://sites.wustl.edu/acag/datasets/surface-pm2-5/*)* [48], which aim for global accuracy at the potential expense of applicability to local and hyperlocal conditions (e.g [49]).

There are innumerable approaches to creating such derived datasets, which have different benefits and drawbacks and rely on different assumptions, leading to potentially different outputs for what are ostensibly the same quantities. The variety of such derived air quality datasets can be a strength or a weakness. On the one hand, it can be difficult to know which dataset is suitable for a particular region or application, versus those that might have biases or make assumptions that are unsuitable. On the other hand, if these datasets are openly available and interoperable, it may be possible to quickly test multiple datasets to find the one best suited to a particular use or to use several datasets via an ensemble approach (e.g [50]). It is also important to have access to other open data, especially in-situ measurements, both for developing new and validating existing derived datasets; the lack of such open data will bias derived datasets towards a better representation of regions with more open data.

### Data Repositories

Data repositories where users can contribute data and metadata (i.e., descriptive narratives or references accompanying the air quality data) are a go-to resource for many air quality researchers. Table 2 shows some examples of platforms that aggregate and share air quality data. Many of them allow user contributions; others perform the search of air quality data sources themselves.

In addition, many air quality researchers use generic open data repositories like Zenodo (https://zenodo.org/), osf.io, or other online data commons that host any type of dataset (not just air quality) to provide their datasets publicly [51–54]. For example, a project called SensEURcity (“a multi-city air quality dataset collected for 2020/2021 using open low-cost sensor systems”), which involves open-source sensors (developed by the involved research groups), provides their data and metadata publicly and freely through *Zenodo*, an online platform funded by CERN, OpenAIRE and the EU [55]. Similarly, the QUANT project [52] has made its multi-year, high-resolution measurements (both reference-grade and sensor data) and metadata available through *CEDA *(https://catalogue.ceda.ac.uk/) for 14 commercially available sensor brands [56].

**Table 2:** Some examples of third-party platforms that aggregate and share air quality data and metadata

Organization or entity	Advantages	Disadvantages	Website URL	Description and Use Cases
TOAR	Global coverage, data validated through IGAC TOAR activity	Sparse coverage in LMIC	https://join.fz-juelich.de/index.html	Aggregates long-term ozone measurements at the surface until the tropopause Data are from ground-level monitoring stations, satellites, aircraft Also hosts model data
OSCAR	Summarizes capabilities of international observing systems	Data are still separated across many repositories	https://space.oscar.wmo.int/	Aggregates user-defined requirements for observations of physical variables related to application areas of the World Meteorological Organization (WMO)
OpenAQ	Provides global data and metadata in a unified format	Limited to sources providing open data	https://explore.openaq.org/	Aggregates and harmonizes open air quality data from across the globe onto an open-source, open-access data platform
sensor.community	Based on open sensing technology	Coverage focused in Europe	https://sensor.community/	Community-driven, open environmental data where users can submit air quality data from DIY sensors
AQICN	Integrates data from many national networks	Data use different AQI scales, limiting comparability	https://aqicn.org/	Aggregates real-time global air quality information (air quality indices)
canair.io	Based on open sensing technology	Global coverage is unclear	https://canair.io/	Citizen science project using mobile and static sensors to measure air quality with cell phones and low-cost technology

Some data repositories more specifically serve data related to air quality. For example, the World Meteorological Organization’s (WMO) *OSCAR* (https://space.oscar.wmo.int/) is a “resource developed by WMO in support of Earth Observation applications, studies and global coordination.” From here, quantitative, user-defined requirements for observation of physical variables such as those related to weather, water and climate can be accessed. Specific information on all earth observation satellites and instruments, and expert analyses of space-based capabilities may also be accessed from OSCAR. The *Tropospheric Ozone Assessment Report (TOAR) *(https://join.fz-juelich.de/index.html), provides access to long-term ozone measurements from over 10,000 stations around the world as well as satellite, aircraft, and model data [57]. All data are freely accessible for research and have been used across air quality and climate change assessments at national and global scales [58, 59].

### Visualization and Analysis

Finally, there are openly available online platforms for visualizing air quality information. These are often created by the data providers or aggregators, with the goal of facilitating accessibility of their data, especially for those without the expertise or infrastructure needed to work directly with the raw data. For example, open platforms for satellite data visualization include *Worldview* (https://worldview.earthdata.nasa.gov/), *Giovanni*(https://giovanni.gsfc.nasa.gov/giovanni/), *VEDA*/*JPL data mapper* (https://www.earthdata.nasa.gov/esds/veda) (https://ideas-digitaltwin.jpl.nasa.gov/airquality/dat/#b=BlueMarble_ShadedRelief_Bathymetry%26d=2018-12-01%26l=acag-v5gl01-gwrpm25_monthly_PM25(1)%26vm=2D%26ve=-142.6772114429744,18.046616163851596,-52.67721144297441,62.273178663851596%26pl=true%26pb=true%26c=false%26tr=false%26tlr=months), *FIRMS* (https://firms.modaps.eosdis.nasa.gov/map/), *AerosolWatch* (https://www.star.nesdis.noaa.gov/smcd/spb/aq/AerosolWatch/), *JSTAR Mapper *(https://www.star.nesdis.noaa.gov/mapper/#zoom=4/date=2024Sep26/lat=39.5/lon=-98.4/tcon=true/granon=false/bordon=true/view=asc/l1on=false/satval1=SNPP/sensval1=Land/prodval1=efrp/levval1=null/op1=1/l2on=false/satval2=SNPP/sensval2=Land/prodval2=efrp/levval2=null/op2=1/l3on=false/satval3=SNPP/sensval3=Land/prodval3=efrp/levval3=null/op3=1) *RAMMB/CIRA* (https://rammb-slider.cira.colostate.edu/?sat=goes-16%26sec=full_disk%26x=10848%26y=10848%26z=0%26angle=0%26im=12%26ts=1%26st=0%26et=0%26speed=130%26motion=loop%26maps%5Bborders%5D=white%26p%5B0%5D=geocolor%26opacity%5B0%5D=1%26pause=0%26slider=-1%26hide_controls=0%26mouse_draw=0%26follow_feature=0%26follow_hide=0%26s=rammb-slider%26draw_color=FFD700%26draw_width=6), *EUMETSAT viewer* (https://view.eumetsat.int/productviewer?v=default) and *CAMS* (https://atmosphere.copernicus.eu/); these resources provide visualizations of data from various missions relevant to different aspects of air quality, along with the ability to perform basic analyses or comparisons. The US EPA provides *AirNow*(https://gispub.epa.gov/airnow/), a popular tool for air quality information, along with the *Fire and Smoke Map* (https://fire.airnow.gov/). Many companies that provide dashboards for their air quality instruments also provide data visualization capabilities, often relying on publicly available data.

A key advantage of these platforms is increasing the accessibility and usability of data by non-expert users, as well as allowing experts to easily browse databases in search of events of interest for further analysis. A risk of such platforms, however, is that their simplified representations of data may obfuscate nuances, leading casual users to incorrect conclusions. The balance between accessibility and clarity of data representations is an area of ongoing development and dialogue between the data experts and their user communities.

### Challenges

Many of the challenges associated with open air quality data relate to accessibility, whether due to technical, infrastructural, legal, or institutional barriers. In this section, we examine key barriers to access and use of open data, including: (1) limitations in data generation due to resource and capacity constraints, which results to geographical disparities in coverage; (2) data sharing practices and legal or institutional restrictions; (3) heterogeneity in data types, formats, and applicability; (4) limited computational infrastructure; (5) gaps between outdoor data and real human exposure; and (6) concerns around data misuse. While not all of these challenges are strictly about data access, they significantly affect the ability of users to fully benefit from open air quality data platforms, with disproportionate impacts in regions with fewer resources.

#### Data Generation

A potential barrier to collecting air quality data is the maintenance needs of reference-grade monitors. Specifically, maintaining such monitors is hindered by limitations in access to necessary resources, supplies, and spare parts, coupled with the requirement for specialized technical skills. These barriers may be overcome by leveraging international expertise and partnerships. Intercountry and local government air monitoring efforts, as well as air monitoring projects by non-governmental organizations (such as academic institutions, community-based organizations, civic groups and citizen scientists), are also increasing worldwide [13]. For example, some countries participate in a regional intergovernmental effort that includes monitoring, such as the International Centre for Integrated Mountain Development (ICIMOD, https://servir.icimod.org/thematic-focus/air-quality/) which serves the Hindu Kush Himalaya region and Air Quality Central Asia (https://aqcaplatform.asia/about) which supports monitoring in Kazakhstan, Kyrgyzstan, Uzbekistan and Tajikistan. Breathe Cities (https://breathecities.org/) is a combined initiative of a growing number of global cities to combine data from stationary and mobile air sensors with grassroots campaigns to build awareness of air pollution and support municipal action. One of the cities is Accra, and their data is made available through https://breatheaccra.org.

#### Data Sharing

There are reasons why sharing data might not be seen as beneficial. For example, Ivey et al. mention that traditional academic practices become problematic when researchers pursue “helicopter” or “parachute science,” where data from communities are published without representation or validation of accuracy from the community being studied [25] or the government authority operating the monitors. These practices can lead to data being preemptively closed and unavailable to the public, but can also lead to hampering action that benefits environmental and public health. Another reason is the threat to data privacy and security: as data platforms increasingly incorporate real-time hyperlocal data (e.g., from sensors or monitors in communities), issues around data privacy and security become significant, requiring actions to protect sensitive information while maintaining openness.

When data sharing is warranted, however, upstream mandates requiring open data with specific stipulations could be influential and necessary for open data access. For example, in the United States, projects receiving federal funding are mandated to provide accessible data. One example of a platform that results from such mandates is the Climate and Health Outcomes Research Data Systems (CHORDS) platform of the National Institute of Environmental Health Sciences: (https://chordshealth.org/*).* The European Union (EU) also requires its member states to provide up-to-date ambient concentrations of various pollutants [60]. One example of a portal from the EU is https://aqportal.discomap.eea.europa.eu/. In addition, there are also philanthropic organizations that specify and outline definitions of “openness” [61, 62]. However, funding limitations and platform sustainability could be put in jeopardy when grant funding or governmental support, which may not be permanent, is not sustained. For some countries, monetizing the data is a way to raise funds. This poses a risk to the long-term storage and availability of air quality data, especially in regions where funding or monetization schemes are limited. In addition, long-term data storage can be both a logistical issue and dependent on the current state of government affairs. For example, in early 2025, public access to the US EPA EJScreen, a tool used to explore disparate pollution impacts and was in operation since 2015 to explore disparate pollution impacts, was discontinued. Likewise, the NASA SERVIR program, a partnership between NASA and the United States Agency for International Development (USAID), provided support for international dashboards but was removed from NASA websites after funding freezes and layoffs at USAID. The US Department of State’s global air quality program which measured air quality at US embassies and consulates around the world and made the information freely and publicly available, also was cut back and currently, data are no longer publicly accessible. During the time that the program was active, the advancements from it demonstrated success in improving air quality action in countries with limited monitoring and helped saved money for the US State Department and the cities that hosted the monitors [63–65].

#### Disparate Data Types, Formats, and Applicability

Overlapping data from different sources can make it difficult to tell what dataset to use in what situations. In these cases, flowcharts like what NASA HAQAST provides can be very useful (see https://haqast.org/data-and-tools/). Large datasets can be difficult to download and manipulate and can be offered in formats unfamiliar to health practitioners and researchers. How remotely sensed data relates to surface air quality may be unclear in some instances, e.g. in what conditions can aerosol optical depth (AOD) be used as a proxy for ground PM_2.5_? Models can have bias errors, especially when compared to hyperlocal air quality. For air quality monitoring required by regulation, air quality data collected may still be widely variable in terms of data accuracy, spatial distribution, and temporal frequency. Harmonization may or may not fall into the responsibilities of the complying body. Some governments may develop and impose data formats and data standards to reduce barriers to access. In addition, “data intermediaries” i.e., third-party aggregators and harmonizers, can benefit the community by performing harmonization [66].

#### Computational Power

Users might face challenges in the implementation of complex air quality models. Effective models might be constrained by the need for robust infrastructure and sufficient computational power, which might not be accessible to users unaffiliated with research institutions. For example, many users who only rely on personal and laptop computers may lack the computing resources needed to work with large datasets. Easy-to-use platforms (e.g., NASA Worldview (https://worldview.earthdata.nasa.gov/) and Giovanni (https://giovanni.gsfc.nasa.gov/giovanni/)) can allow users to work with datasets without having to download and manipulate large files.

#### Exposure Misclassification

In-situ, satellite-derived, and modeled data are almost exclusively measurements or estimates of outdoor air concentrations and often at large spatial scales. However, air pollutant concentrations can vary widely across distances and outdoor concentrations are not always representative of those experienced indoors. In general, air pollutant concentrations are lower indoors compared to outdoors, but can be elevated, often dependent on occupant behavior and length of time spent indoors[67]. For example, Chambliss et al. found concentrations of ultrafine particles measured by mobile monitoring sites to be more than twice as high as concentrations provided from land-use regression models [49]. Rosales, et al. found that high number concentrations of sub-10-nm particles were produced from the use of surface cleaners indoors, resulting to exposures comparable to or exceeding that of ambient exposure to vehicle exhaust particles [70]. In LMICs in particular, indoor exposures to air pollution can be quite high in areas where solid fuels are used for household cooking and heating and contribute substantially to the overall disease burden [68]. When using open data containing concentrations of outdoor air pollution, researchers, policymakers, and the public should be aware that these concentrations are not always reflective of actual exposures.

#### Misuse and Determining Appropriate Levels of Use

 Assessing what level of rigor and accuracy is necessary for different purposes and objectives is helpful such that potential negative impacts from the misuse of data (e.g., non-validated data) can be minimized. The requirement of sensor assessment and data validation will depend on the intended use of the data, for example, data that are used for increased public understanding (such as community engagement and education) will not be necessary to undergo as much quality assurance as data that are used for legal and policy actions, such as regulatory standard setting and enforcement [69]. Post-processing algorithms may or may not be necessary depending on the purpose. Open data availability impacts conversations and policy action on air quality.

### Perspectives and Future Recommendations

When thinking about “openness”, data providers should be guided by a framework that allows an open data platform to increase its impact in data-scarce regions, such as in LMICs. Third-party aggregators and data intermediaries can also help ensure that platforms that serve data-scarce regions equitably can exist. Addressing gaps in technical training can make open data impactful.

Ultimately, in-situ “ground truth” data will always be needed. Efforts should be made to support existing monitor networks with infrastructure, resources, and local technical capacity building, as well as to expand and supplement these networks to close global data gaps, and finally, to encourage and support openness for the collected data. Support for such efforts should be international in scope, considering the global public good provided by these data. However, efforts should be locally directed to ensure that community needs are being met and local conditions are taken into consideration. To further enhance the utility and accuracy of satellite data, there is a critical need to expand ground-based measurement networks *(e.g.*, AERONET and PANDONIA), particularly in LMICs, to support robust validation of satellite-derived products. Additionally, the current predominance of geo-stationary satellite missions in the Northern Hemisphere highlights a geographical disparity that limits their applicability to other regions [9]. International multilateral agencies should consider funding projects aimed at expanding this coverage, which would benefit the entire world, especially considering that entangled issues like air pollution and climate change require a global perspective. Existing global networks can be leveraged to increase capacity (e.g., enabling local managers and citizen science groups to effectively use its data).

Simple and accessible tools are needed to bring together multiple sources of information and easily make appropriate comparisons between them. Co-development of these tools between subject matter experts and data users is essential to ensure they provide trustworthy, actionable information. The tools themselves, as well as their development, should follow open science principles.

### Key References

World Meteorological Organization (2024) Integrating Low-cost Sensor Systems and Networks to Enhance Air Quality Applications. GAW Report No. 293 https://library.wmo.int/idurl/4/68924.

World Health Organization (2023) Overview of methods to assess population exposure to ambient air pollution https://www.who.int/publications/i/item/9789240073494.

Van Beveren R (2024) Environmental decision makers have a data plumbing problem. Data intermediaries can help. https://www.policyinnovation.org/blog/dataintermediaries?mc_cid=54f8893a22.

Duncan BN, Malings CA, Knowland KE, Anderson DC, Prados AI, Keller CA, Cromar KR, Pawson S, Ensz H (2021) Augmenting the Standard Operating Procedures of Health and Air Quality Stakeholders With NASA Resources. GeoHealth 5:e2021GH000451.

United States Government Accountability Office (2024) Air Quality Sensors: Policy Options to Help Address Implementation Challenges. https://www.gao.gov/products/gao-24-106393

## Data Availability

No datasets were generated or analysed during the current study.
